# Conductance-Based Interface Detection for Multi-Phase Pipe Flow

**DOI:** 10.3390/s20205854

**Published:** 2020-10-16

**Authors:** Shiyao Wang, Jesus Leonardo Corredor Garcia, Jonathan Davidson, Andrew Nichols

**Affiliations:** 1 Department of Civil and Structural Engineering, The University of Sheffield, Western Bank, Sheffield S10 2TN, UK; swang132@sheffield.ac.uk (S.W.); jlcorredorgarcia1@sheffield.ac.uk (J.L.C.G.); 2 Department of Electronic and Electrical Engineering, The University of Sheffield, Western Bank, Sheffield S10 2TN, UK; jonathan.davidson@sheffield.ac.uk

**Keywords:** electrical conductance sensor, multi-phase interface detection, medium electrical conductivity measurement, low-cost sensor

## Abstract

Sediment and flow depth monitoring in sewers is important for informing flow models and for predicting and mitigating against sewer blockage formation and surcharge. In this study, a novel sensor based on conductance measurement has been developed and tested under a laboratory environment and validated by a finite-element model. The relative conductance is measured between pairs of adjacent electrodes to provide a conductance profile along the sensor length. A piecewise linear relationship between conductance and electrode length was derived and the interface positions between sediment, water, and air can be determined from the profile. The results demonstrated that the root mean square error of the model and the measured interface level are within 1.4% and 2.6% of sensor’s measurement range. An error distribution of interface height shows that all anticipated errors are within the resolution of the electrode length increments. Furthermore, it was found that the conductivity of the measured medium is proportional to the gradient of the linear relationship of conductance and electrode length. It could therefore prove a valuable new tool for the accurate quantification of sediment and flow levels in sewer conduits, coastal environments, drainage systems for transport networks, and other industrial or academic applications.

## 1. Introduction

Sewers function as collectors and transporters for wastewater and stormwater, but a significant proportion of sewer solids can settle and remain in sewer pipes [1]. Sediment deposition in sewers decreases the capacity of pipes and increases the risk of sewer overflows during high flow events, while also influencing many physical, chemical, and biological processes that can generate harmful gasses and corrode the inner pipe wall [2]. Sediment transport, deposition, and entrainment also influence the treatment processes at wastewater treatment plants (WWTP) and the quality of receiving waters, particularly during overflow [3]. These problems can increase maintenance and safety costs and bring potential health and environmental issues. Sediment management is a challenge for sewer managers; for example, in Nantes, 2500 tonnes of sediment deposits are removed from sewers annually because sewer sediment is seen as a primary factor in sewer overflows while increasing the risk of pipe damage [4]. Moreover, sewer solid deposition and transformation are complex processes, as sewage is a mixture of minerals and organic materials [5]. Hence, research into low-cost and long-term sediment monitoring is necessary to improve understanding of the sedimentation process [6] and enable real-time management approaches.

Although the automatic measurement of sediment depth has been attempted since at least the 1990s [7], techniques for in situ real-time measurements of sewer deposits remain limited. Several previous attempts have addressed the measurement of sediment and flow from different perspectives and using various principles. For example, acoustic technology is one of the most common methods for measuring the morphology of sediment deposits in large-scale sewers [8,9,10]. In sewers, acoustic measurement devices are usually floated on the liquid surface and calibration is necessary at the beginning of the measurement. When sensors float on a free surface, the position of the sensors changes during measurement and reduces the accuracy [11]. They also present an obstruction to the flow and can collect unwanted debris. Some acoustic devices can also measure the profiles of the flow bed with a rotated sonar; these devices measure without any disturbance of the sediment through analysing the attenuation and transit time of acoustic waves [8]. However, a series acoustic measurement system usually includes several component devices, which means high cost and energy consumption and complicated manual operation during measurement.

Some sensing methods based on the sediment material’s electrical properties have been developed for the quantitative monitoring of coastal, fluvial, or sewer environments. Wang and Jiang [12] placed vertically distributed multi-anode sediment microbial fuel cells (SMFC) in a water–sediment interface for real-time monitoring of the sediment height. The array anodes span from below the water–sediment interface to above the interface, while a cathode is within the water phase. The height of each anode is fixed during the setup. When an anode is below the water–sediment interface, the voltage would increase to 40 mV, and otherwise, the voltage is 0. Each anode is paired sequentially with the cathode to determine which anodes are below the sediment interface. However, the measurement range of the sediment height is limited (6 cm) and the water level cannot be determined. The other limitation of this method is that it is binary (40 mV or 0), which means that it can only detect which anodes are nearest to the sediment interface but cannot determine the actual interface height. Thus, the resolution of the sensor depends on anode spacing.

Ridd [13] proposed for coastal applications a thin rod with ring electrodes that can monitor the sedimentation process based on the difference in electrical conductivity of seawater and saturated sediment. The resolution of this sensor is limited to the electrode spacing. De Rooij [14] developed a device consisting of an array of electrodes on the base of a tank, connected via the filled water to a reference electrode located within the fluid. Changes in the depth of sediment between the reference and bed electrodes caused a change in reference resistance. Although the result is accurate, the instrument required a precise calibration for a given water depth and conductivity of the medium, and any fluctuation in either of these parameters would significantly affect the accuracy of the readings. Jansen et al. (2005) [15] developed and tested a mobile device that measured resistivity of marine sediments and, with the help of pressure sensors, could estimate the deposit depth; however, its complexity rendered it unsuitable for small scale applications. Li et al. [16] and Tollefsen et al. [17] used capacitance to measure sediment concentrations in water and multiphase flows (water, gas, and oil), respectively; although both studies reported high accuracy, the applications do not provide meaningful insights to sedimentation processes because the formulation at the heart of the sensing process relied on multiple parameters not easily measured in sewer flows, such as flow salt content and soil types. Besides, a calibration process is necessary for these methods because the performance is not fixed for different test materials.

Some methods also use electrical resistance tomography technology to develop a visual representation of sediment behaviour within sewers. Schlaberg et al. [18] used a U-shaped sensor array to measure the concentration and deposition of suspended sediment based on the conductivity of the medium. Layers with different conductivity can be detected, but the sensitivity of the U-shaped sensor is not uniform, which leads to anomalies in the resulting tomographic image. Electrical capacitance tomography and electrical resistance tomography are used to detect interfaces in other relevant areas, such as gas–solid fluidised beds [19], measuring the particle concentration within moving suspensions [18], and imaging the distribution of different conductive media [20]. However, tomography technology is relatively computationally expensive and sensitive to the setup, and also has high power requirements [21] that can render it impractical for a remote field device.

Though conductivity sensors are rarely used for monitoring sewers, electrical conductivity is an important parameter that can enable an analysis of the structure and properties of sediment or soil. Sophocleous and Atkinson [22] designed a thick film conductivity sensor by using four gold-stripe electrodes to measure the conductivity of soil with a 100 Hz to 5 kHz swept frequency range. This sensor is cost-effective and produced a good linear relationship between the output voltage and the conductivity of measured samples. Li et al. [23] used five layers of carbon mesh anode and a carbon air cathode inserted into soil to measure the impedance of soil and detect the relationship of conductivity and moisture content of the soil.

The previous studies have illuminated the potential for conductance-based methods to characterise interface levels and media composition; however, a sensing platform has never been developed that enables deployment of conductance methods in a sewer environment at low cost and power. In this study, a novel multi-electrode sensor based on electrical conductance measurements is proposed for synchronous, near-instantaneous monitoring of the depths of sediment and water in a sewer pipe. Firstly, the structure and the analytical theory behind the sensor are introduced in Section 2. Then, Section 3 presents the methodology for experimental and finite-element model investigations. Section 4 compares the experimental and finite-element model results. Finally, Section 5 summarises the main conclusions

## 2. Sensor Architecture and Theory

### 2.1. Design of the Conductive Sensor

**Figure 1:**
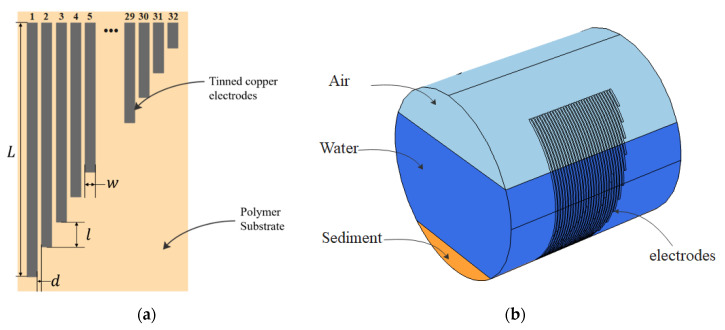
(**a**) Schematic drawing of the conductive sensor; (**b**) 3D view of the arrangement of the sensor and media in a circular pipe.

The structure of the sensor is based on a design by Nichols [24] which can quantify the depths of water and sediment simultaneously. As shown in Figure 1a, the sensor consists of 32 parallel tinned copper electrodes placed on an insulated polymer substrate. The electrodes decrease in length linearly from electrode 1 to electrode 32. In Figure 1a, L represents the length of the longest electrode, w is the width of each electrode, and d and l are the distance between electrodes and the length difference between two adjacent electrodes, respectively. In this paper, because the sensor is considered to be applied in a circular sewer pipe that contains different heights of media, the substrate of the sensor is curved to match to the internal wall of a circular pipe section and the electrodes are exposed to the media on the inside of the pipe, as shown in Figure 1b.

### 2.2. Theory

The theoretical principle behind the sensor is the relationship between the electric field generated between two electrodes and the environment surrounding those electrodes. Hence, it depends upon the inherent electrical properties of the local media, the sensor geometry, and the excitation method. As a first approximation, the media in a sewer pipe or open drainage channel can be divided into three phases: air, water, and saturated sediment. These three media have a significant difference in electrical conductivity. The conductivity of tap water is in the range of 50 to 80 mS/m [25], while air can be considered as an insulating material with negligible conductivity. Saturated sediment has a conductivity lower than that of water and the conductivity depends upon its porosity and permeability [26].

**Figure 2:**
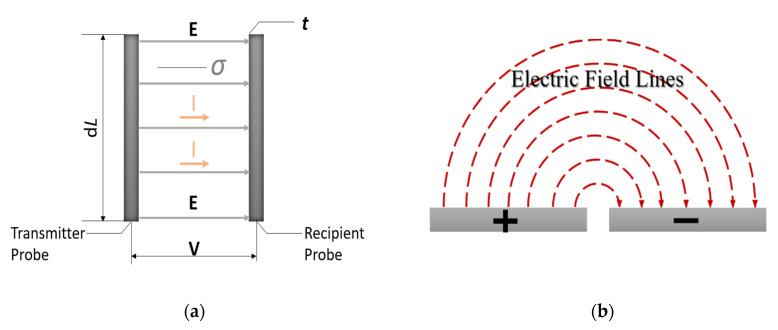
(**a**) Generic section of a typical configuration of pairs of probes. (**b**) Operating principle evolution from parallel capacitor to flat capacitor.

The sensor functions by determining the conductance between every two adjacent electrodes, i.e., electrode pairs. As shown in Figure 2a, E is the electric field generated by the electrodes. The recipient electrode connects to the earth, which leads to an electric charge movement, i.e., a current is generated between the electrodes. I represents the current density in a 2D scenario. However, due to the thickness of the electrodes being much smaller than the width, each electrode pair can be assumed to approximate to two parallel plate capacitors, as shown in Figure 2b. An electrical fringing field is also generated between the anode and cathode, whereby the field extends into the measured medium for a short distance, from the top surface of the electrodes. Since the electrodes’ shape does not change, this effect can be considered as a constant factor increasing the apparent conductivity of any medium. According to Ohm’s law, I =GV, when a fixed voltage (V) is applied across two electrodes and the current (I) between them is measured, the conductance (G) is proportional to the current (I). Conductance is proportional to the area of the conducting medium (A) and has an inverse relation with the distance between the electrodes (l). For the proposed sensor, the area of the conducting media (A) is equal to the product of extension of the differential electrode’s length (dL) and thickness (t), which is perpendicular to the paper, as shown in Figure 2a. In this study, it is difficult to measure the area (A) due to the fringing field, but A/l must be proportional to dL, which is known during the design process. Simultaneously, the medium is assumed to be homogeneous, i.e., the fringing electric field is uniform along the electrode. Hence, the conductance (*G*) is still proportional to the conductivity (σ) multiplied by the electrode’s length (dL) as follows:

(1) $$G\propto \sigma \frac{A}{l}=\sigma \frac{t\times dL}{l}\propto \sigma dL$$

**Figure 3:**
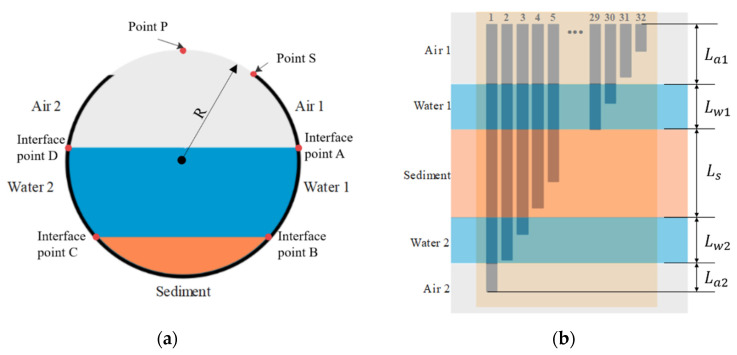
(**a**) Cross section view of the sensor attached on the pipe inner wall; (**b**) five-phase media along the sensor.

**Figure 4:**
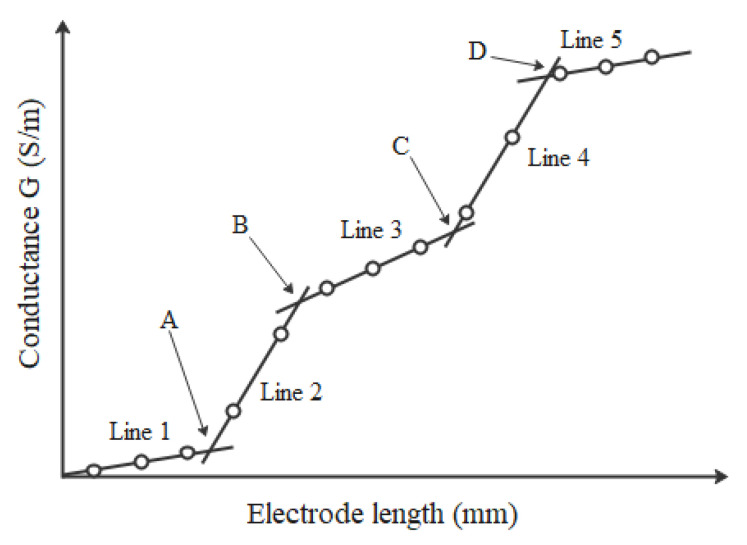
Linear piecewise relationship between conductance and increasing length.

In this study, the application is considered in a sewer pipe containing three layers of media: sediment, water, and air. The media in the sewer pipe can be considered as five phases along the length of the electrode array, as the sensor runs down one sidewall and up to the opposite side, as shown in Figure 3b. To simplify the analytical theory, the conductivity of each medium is considered fixed and homogeneous. Hence, the conductance of the sensor submerged into the five-phase media is expressed as:

(2) $$G\propto \sigma_{a}L_{a1}+\sigma_{w}L_{w1}+\sigma_{s}L_{s}+\sigma_{a}L_{a2}+\sigma_{w}L_{w2}$$

where $\sigma_{a}$, $\sigma_{w}$, and $\sigma_{s}$ are the electric conductivity of air, water, and sediment;$\;L_{a1}$,$\;L_{w1}$, $L_{s}$, $L_{w2}$, and $L_{a2}$ are the electrode lengths in air 1, water 1, sediment, water 2, and air 2, as labelled in Figure 3. This is a piecewise equation as follows:

(3) $$G\propto \{\begin{array}{c} \sigma_{a1}dL\\ \sigma_{a1}L_{a1}+\sigma_{w1}\left(dL-L_{a1}\right)\\ \sigma_{a1}L_{a1}+\sigma_{w1}L_{w1}+\sigma_{s}\left(dL-L_{a1}-L_{w1}\right)\\ \sigma_{a1}L_{a1}+\sigma_{w1}L_{w1}+\sigma_{s}L_{s}+\sigma_{w2}\left(dL-L_{a1}-L_{w1}-L_{s}\right)\\ \sigma_{a1}L_{a1}+\sigma_{w1}L_{w1}+\sigma_{s}L_{s}+\sigma_{w2}L_{w2}+\sigma_{a2}\left(dL-L_{a1}-L_{w1}-L_{s}-L_{a2}\right) \end{array}\begin{array}{l} \left(dL\leq L_{a1}\right)\\ \left(L_{a1}<dL\leq L_{w1}+L_{a1}\right)\\ \left(L_{w1}+L_{a1}<dL\leq L_{w1}+L_{a1}+L_{s}\right)\\ \left(L_{w1}+L_{a1}+L_{s}<dL\leq L_{w1}+L_{a1}+L_{s}+L_{w2}\right)\\ \left(L_{w1}+L_{a1}+L_{s}+L_{w2}<dL\leq L_{w1}+L_{a1}+L_{s}+L_{w2}+L_{a2}\right) \end{array}$$

which shows that the determined conductance (G) and the electrode length (dL) have a linear relationship for each sub-domain of Equation (3) (i.e., for each medium), and the gradient of each line is proportional to the conductivity of the medium. The conductance is measured from each pair of adjacent electrodes. According to Equation (3), the length and conductance of each electrode pair are plotted, as shown in Figure 4. Lines 1, 2, 3, 4, and 5 correspond to the air 1, water 1, sediment, water 2, and air 2, respectively, and points A, B, C, and D match to the interfaces. The height of the water layer and height of the sediment layer can thus be determined from the interface positions A, B, C, and D.

In this study, a MATLAB algorithm was developed based on Optimal Piecewise Linear Regression Analysis (OPLRA) and Fuzzy Piecewise Linear Regression (FPLR) methods to determine the best linear fitting equation. The FPLR method builds upon the concepts of fuzzy sets to yield a possibility model, which instead of being a single segmented function describing the desired behaviour, actually yields a segmented region of possibility bounded from below and above by two optimal solutions to a Mixed Binary Integer Programming [27]. The OPLRA method uses linear programming to find the set of segmented linear functions that yields the lowest absolute error in a piecewise linear regression [28]. After collecting all 31 conductance results from 31 electrode pairs, the algorithm allocates them to several groups in order and performs linear regression to each group. The number of groups depends on the number of segments in the piecewise function. Because there is no prior information regarding the partitions and their probability, it is assumed that all possible partitions that could be obtained by distributing the number of points into the number of groups have the same probability. The linear regression is solved using a polynomial, i.e., least square fit. The algorithm performs linear regressions to all possible groups and calculates the standard deviations of the points in each segment. Finally, the group with the minimum average of standard deviations of all segments was selected.

The intersections of adjacent segments can be determined from the slope and intersect values of the regression lines. To prevent the procedure forming degenerate groups, each group must contain at least two data points. The computed intersections should lie between the endpoint of the previous segment and the first point of the following segment.

To obtain the piecewise fitting line, the values of conductance associated with each pair of electrodes must be related to a specific length. Since the electrode lengths in a given electrode pair are different, each electrode pair is identified with the average length of the two electrodes.

Since the sensor is attached to the inner pipe wall, the positions of intersection points are on the circle’s circumference. The depth (h) of each phase can be determined using the following equation:

(4) $$h=R\times (1+\cos\left(\frac{L+a}{R}\right))$$

where a is the arc length between sensor’s starting point (point S in Figure 3a) and circle’s top point (point P in Figure 3a); L is the position of the intersection point along the electrodes (i.e., the location of intersection point on the electrode length axis in Figure 4); and R is the pipe radius. Because the sensor was manually fixed to the pipe surface, the distance length between points A and P is known.

For an angled sediment layer, if the two intersect point depths $\left(h_{1},h_{2}\right)$ are determined by using Equation (4), the mean sediment depth (h) and rotation angle (θ) can be determined by using Equations (5) and (6):

(5) $$\theta =\arctan\left(\frac{\left|h_{1}-h_{2}\right|}{\sqrt{2Rh_{1}-h_{1}^{2}}+\sqrt{2Rh_{2}-h_{2}^{2}}}\right)$$

(6) $$h=R\times (1-\cos\left(\theta +\cos^{-1}(\frac{R-h_{2}}{R})\right)$$

Because the length of each electrode is known from the design process, through determining the linear fitting equation of each sub-line in the piecewise equation, the position of the intersection points can be found. This process requires no calibration.

## 3. Methodology

### 3.1. Parameters for Physical Experiments and Parallel Simulations

In this paper, both experiments and finite-element modelling are used to assess the feasibility and reliability of the sensor in a circular pipe, and the model is then extended to enable an in-depth quantification of minimum expected measurement error (under ideal conditions). In this paper, the length of the longest electrode (L) of the sensor is 540 mm, and the length difference between the adjacent electrodes (d) is 15 mm. The width of each electrode (w) is 2 mm, and the separation between adjacent electrodes (l) is 0.54 mm. A transparent acrylic circular pipe with a 95 mm internal radius (R) was used to represent a sewer pipe section. This is within the range of typical sewer pipe diameters in the UK [29]. The longest electrode was designed to cover around 90% of the pipe’s inner circumference and thus, the sensor can measure a maximum depth of 172 mm in this case. This length was chosen to simplify the installation and leave space at the top of the pipe for access to set up the experimental conditions. In future embodiments, the sensor electrodes could span the entire circumference.

A range of different scenarios would be examined with various depths of water and sediment. Table 1 shows the setup of each test, which were replicated in both the experiment and the corresponding finite-element analysis (FEA) simulations. The tests included three kinds of scenarios for the pipe contents: (i) water and air; (ii) water, horizontal sediment, and air; and (iii) water, angled sediment, and air. It is worth noting that for different scenarios in the pipe, the number of linear segments would be changed in the piecewise plot. For example, if the pipe contains water and air, there would be 3 segments in the piecewise function.

### 3.2. Experiment Setup

**Figure 5:**
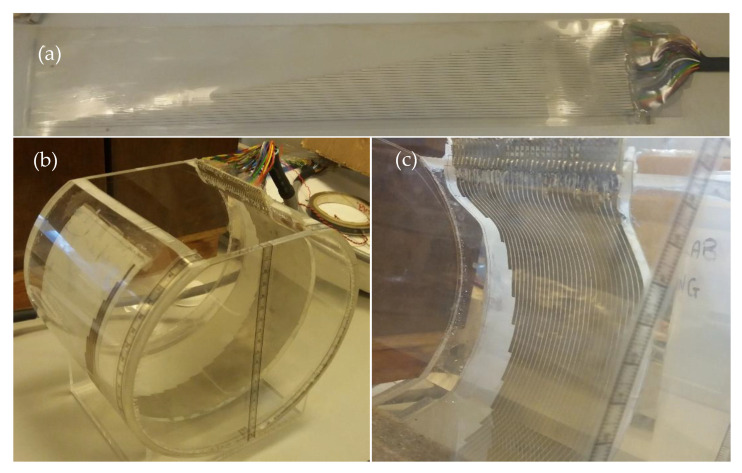
Configuration of sensor and circular pipe used for the experiments: (**a**) the sensor; (**b**) the experiment configuration; (**c**) the sensor attached to the pipe’s inner wall.

For the physical experiments, the sensor was fabricated on a flexible PCB as shown in Figure 5a and was attached to the inner surface of the transparent pipe section, matching the dimensions described in the previous section, as shown in Figure 5b,c The length between the sensor’s starting point and pipe’s top point (i.e., the value of a in Equation (4)) is 45 mm. The lab temperature is maintained at 20 ℃.

In the experiment, circuitry with two multiplexers (MUX A and MUX B, RS Components, Corby, UK) and a LabVIEW program were used to collect and analyse the data. The sensor was energised by a bipolar square wave of 8 kHz frequency and 1 V amplitude. Ten cycles (1.25 ms) of the square wave were sufficient to obtain an accurate measurement for each electrode pair. Collecting data from all 31 electrode pairs can be completed within 0.04 s, meaning that data are collected much faster than the time scale of hydraulic or morphological changes in sewer systems.

**Figure 6:**
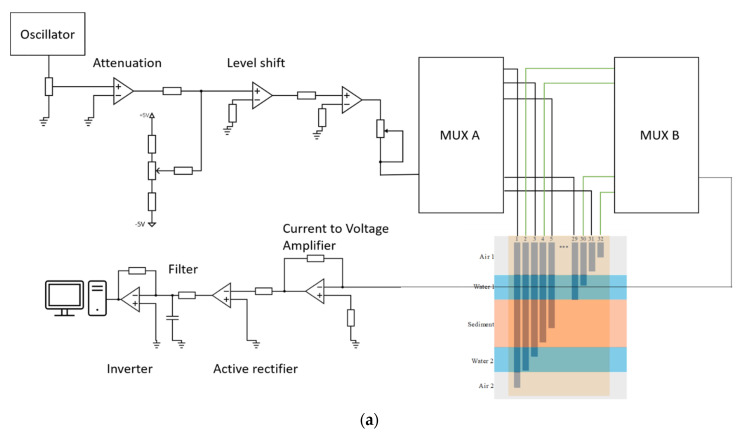

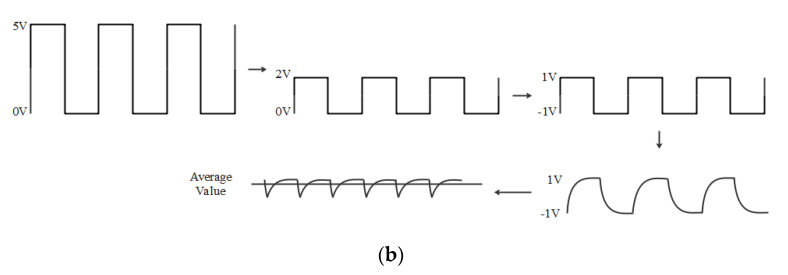
(**a**) Schematic diagram of the simplified measurement circuit; (**b**) Wave form changes in measuring circuit.

In the measuring circuit, MUX A gave the excitation to one of the electrodes numbered 1, 3, 5…29, 31, as labelled in Figure 1a. Meanwhile, the MUX B connected to one of the electrodes numbered 2, 4, 6…30, 32 as a receiver and connected to ground. During the first data collection cycle, the measuring circuit recorded the output square wave from electrode pairs 1–2, 3–4, 5–6…31–32. Then, the second data collection cycle collected the output square wave from electrode pairs 3–2, 5–4, 7–6 up to 31–30. The measurement continually alternated between these two collection cycles. Through a series inverter, filter, and gain circuits, the mean of the absolute value of the current waveform was determined, as shown in Figure 6.

In the experiment, tap water and saturated sand were used to emulate the media in the sewer pipe. The sand size used in experimental testing was 1.18–2.36 mm [30]. The conductivity of saturated sand is variable as it depends on properties such as packing density, porosity, permeability, and tortuosity, so it was measured directly using a HANNA HI991300 waterproof pH, EC, TDS, and temperature meter, which was later used to inform the modelling (Section 3.3 and Section 4.2).

During the experimental test, the depths of water and sediment were set according to Table 1. For the experiments including sediment, water was added first up to a height equal to the required sediment depth; then, the sediment was poured into the pipe until it reached the expected sediment depth. The sediment was gently levelled by hand using a flat scraper to ensure it was level and at the right depth and was left submerged in the water for at least 30 min to ensure that the sediment was fully saturated. Finally, the remaining water was added up to the required water depth, being sure not to disturb the sediment.

It should be noted that the surface of the sediment could not be made perfectly flat. Since the principle of the sensor is to capture the changing points at the pipe perimeter, these points in particular were carefully adjusted to ensure that the sediment–water interface fell close to the points of the perimeter corresponding to the defined sediment depth, to within an estimated error of ±1 mm.

### 3.3. FEA Model of the Sensor

FEA can provide an accurate and effective analysis of the analytical theory and validation of experimental results. In this study, FEA was carried out using COMSOL Multiphysics software (COMSOL Inc., Burlington, CO, USA), which is a finite-element analysis and solver software package for various physics and engineering applications, especially suited to coupled phenomena. For the electrical sediment sensor, the AC/DC module of COMSOL was chosen for analysis, which simulated the sensor’s performance under an ideal environment with 20 °C temperature and at 1-atmosphere pressure. In this model, COMSOL solves a current conservation equation based on Ohm’s law used to compute electric field, current, and potential distributions in conducting media.

**Figure 7:**
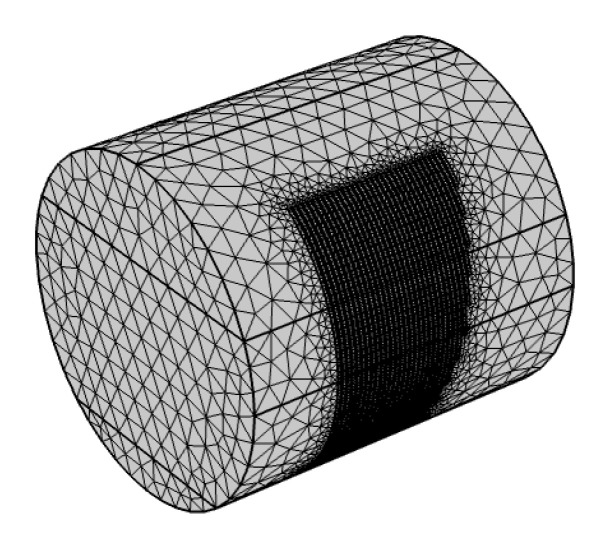
Meshed 3D model of the conductance sensor in COMSOL.

Figure 7 shows a meshed 3D finite element model of the sensor created by using COMSOL. The 3D model was consistent with the experimental setup and used the same geometry as the experimental sensor. The thickness of the electrodes in the model was set at 0 to save computing time. The medium in the pipe was divided into two or three parts set as air, water, and sand according to the experimental setup. The electrical parameters of the media used in the model have been summarised in Table 2, which were measured by the HANNA HI991300 EC meter. In an electrode pair, the longer electrode served as the exciting electrode, which is supplied with a 1V_pk_, 8 kHz sine wave. The shorter electrode is a receiver for current measurement and grounded. The model recorded the output current from COMSOL one electrode pair after another, and the other electrodes were grounded. After solving all 31 electrode pairs, the RMS value of the current wave was determined, then transferred to conductance according to Ohm’s Law. Once the conductance of each electrode pair is obtained, the data analysis method is the same as for the experiment tests described in Section 2.2.

**Figure 8:**
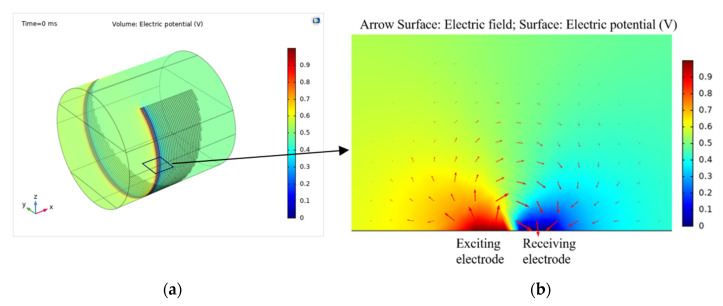
Electric field vectors and electric potential distribution for one electrode pair of the sensor calculated by COMSOL Multiphysics: (**a**) 3D view of electric potential distribution for the model; (**b**) cross section electric field vectors and electric potential distribution of one electrode pair.

Figure 8 shows the calculated electric potential and electric field distributions of one electrode pair. As can be seen, the electric field arrow is from the exciting electrode to receiving electrode and across the media above the electrodes, which also corresponds to the operating principle described in Section 2.2.

## 4. Results and Discussion

### 4.1. Depth Measurement Results

**Figure 9:**
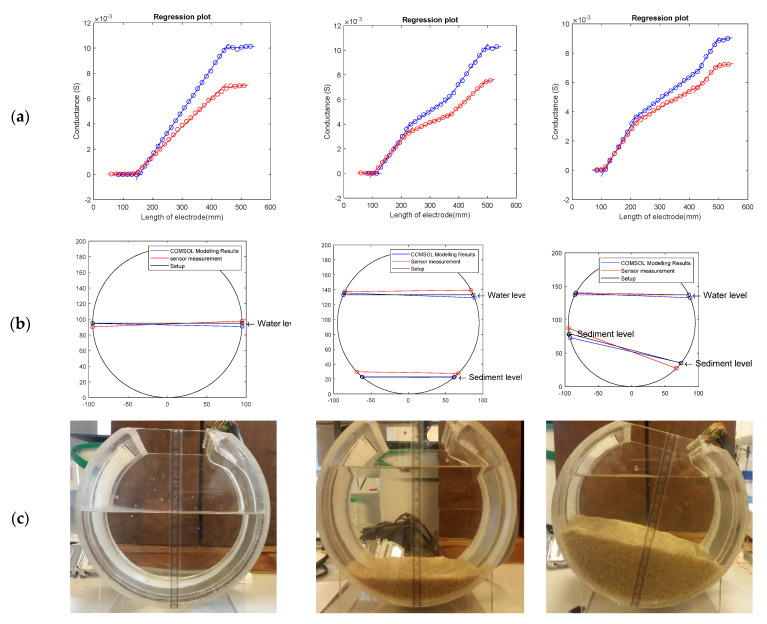
Test W2, S1, and SA1: (**a**) the fitting results of these three tests (blue line: COMSOL modelling results; red line: sensor’s measurement results); (**b**) the calculated depths demonstrated in the circle; (**c**) photograph of the setup in the experiment.

Following the main aim of this paper, to apply the sensor in a pipe environment and measure the depths of water and sediment, it is necessary to compare the depths obtained by the sensor and the model with the actual experimental setup. With mean depth defined as the average height of the left and right interface points, the results and error from the model and the sensor measurements have been compared with the actual depths in Table 3 and Table 4. Since the sand level is not horizontal in the rotated tests (Test Code SA1, SA2, SA3), the depths of the left and right points of the sediment surface are used for validation. As mentioned in Section 3.2, the intersection points of two adjacent lines on the conductance vs. probe length plot represent the interface position between two different media in the pipe. Due to the sensor being attached to the pipe’s inner wall, the probe length corresponds to the perimeter position around the pipe. Once the sensor’s starting position is known, it is a simple geometric problem to transform the perimeter positions of intersection points into a vertical depth. Figure 9 demonstrates the fitting results, determined interface, and the experiment setup of three representative examples—a water-only test (Test Code W2), a water and sediment test (Test Code S1), and a rotated sediment test (Test Code SA1). The interfaces are plotted in a circle to represent the setup scenarios.

Table 3 and Table 4 compare the calculated depths of model and sensor measurements with the actual setup depth. The root mean square error from the model is 2.72 and 2.36 mm for water level and sediment level, respectively, and the root mean square error of the sensor’s measurement is 3.48 and 4.98 mm for water depth and sediment depth, respectively. In other words, the root mean square error of the model and experiment are within 1.4% and 2.6% of the sensor’s measurement range (i.e., the pipe diameter 190 mm) and within 18% and 33% of the electrode tip spacing (15 mm). This shows that the water level measurements demonstrate a higher accuracy than sediment level measurements in the physical experiment and model. The sediment error is greater for the experiment with a bigger root mean square error, though this is likely attributable to the inevitable variability and uncertainty in actual sediment level, which is ±1 mm at the pipe walls, as described in Section 3.2, and the interface is also not perfectly planar.

**Figure 10:**
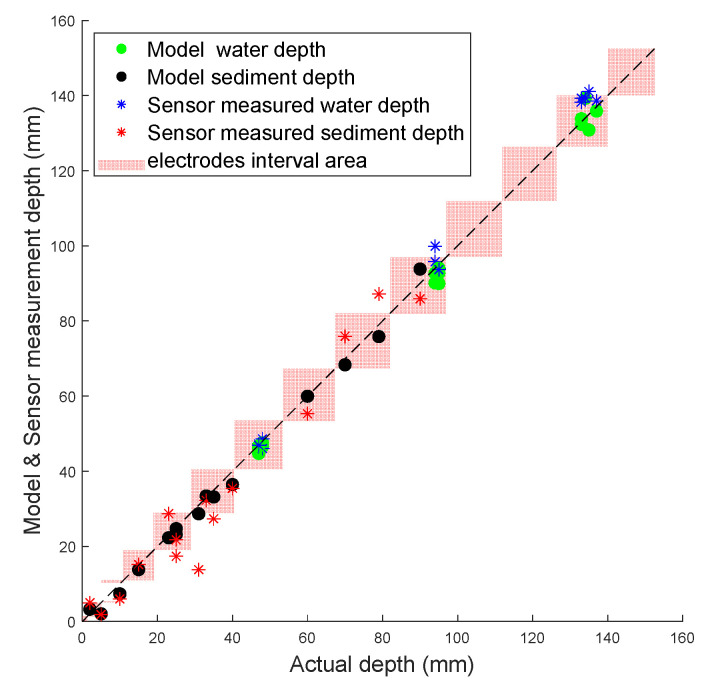
Model and sensor measured results with acceptance range.

The error of the sensor can be influenced by the resolution of the sensor, namely the length of the stepwise increase in electrode length, which is 15 mm for this sensor. Thus, an error within the resolution of the sensor (±7.5 mm) may be considered the worst case, i.e., the interface point on the circumference should be detected to the nearest electrode tip or better. Furthermore, as the sensor was installed within a circular pipe, this perimeter error translates to a smaller depth error near the crown and invert of the pipe (similar in principle to an inclined manometer). Hence, the acceptance range is the height difference between the two electrodes closest to the interface. Figure 10 shows a plot displaying the actual depth vs. the modelled and sensor-measured depth, and the red area is the acceptance range based on the sensor’s physical resolution. It was found that all the model results were within the sensor’s resolution area, and there were just four experimental results outside of the red area. The 45° line in the plot indicates the ideal response.

### 4.2. Sensor’s Resolution Analysis

**Figure 11:**
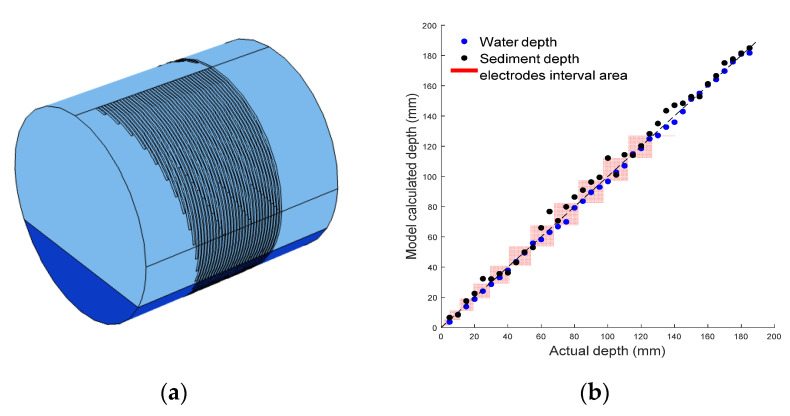
Electrodes fully surround 3D model (**a**). Model results with acceptance range (**b**).

To gain a deeper understanding of the potential accuracy of the sensor, a circular pipe with a fully surrounding electrode array has been modelled, as shown in Figure 11a. The model included 40 electrodes with a 15 mm length difference between adjacent electrodes (the same resolution as used in the previous model and experiment). Figure 11b shows the actual depth vs. the model calculated depth, and the red area represents the sensor’s resolution area. In the model, the level of water was increased from 5 to 190 mm in 5 mm increments. Then, the water was held at 190 mm and the level of sediment was increased from 5 to 190 mm in 5 mm increments. From the figure, most points are seen to be within the red acceptance area, with only six points outside the red area. The root mean square error of water depth and sediment depth are 2.01 and 3.38 mm, respectively. The error of water depth and sediment depth is 1.1% and 1.8% of the sensor’s measurement range, i.e., the diameter of the pipe. Meanwhile, the error of water depth and sediment is 13.4% and 22.5% of the electrode’s length difference, respectively. It was found that the root mean square error in sediment depth is higher than the error (1.37 mm) in water depth. This is because the electrical properties of sediment and water have a significantly smaller difference than that of air and water. This leads to the intersection being less pronounced, and thus, influences the classification of points by the multiple linear regression algorithm.

### 4.3. Slope and Electrical Conductivity

According to the theoretical equations in Section 2, the electrical field crosses through the top surface of the electrode pairs, and thus, the gradient of each segment referred to Figure 9a is proportional to the electrical conductivity of the media. Table 5 summaries the mean gradients of each medium in the model and experiment, across all the scenarios described in Table 1, and compares them with the measured conductivity values. It was found that the mean gradient of air is close to zero as expected, which is negligible. As for the model, the mean gradients for water and sediment are 17% and 15% larger than the meter measured water and sediment conductivities, respectively. The sensor-measured mean gradients of water and sediment are 17% and 27% smaller than the EC meter-measured water and sediment conductivities. As mentioned in Section 2.2, due to the influence of the fringing electric field, the calculated conductivity is only expected to be proportional to the media conductivity. From the table, the scaling factor has been calculated. The ratio of actual conductivity and model calculated conductivity in water and sediment are similar, which provides a good basis of the theoretical prediction. The ratio of actual conductivity and experiment-calculated conductivity are slightly different for water and sediment, which can result from the non-uniform electrical properties of the media, especially for the saturated sand. From the piecewise plot in Figure 9a, for media like water and saturated sediment, which have a difference in electrical conductivity, the sensor can demonstrate this difference in gradients. Hence, in addition to interface monitoring, this sensor can be put forward for monitoring changes in composition that affect conductivity, for example salinity of the medium, sediment compaction, or microbial activity.

### 4.4. Limitation

**Figure 12:**
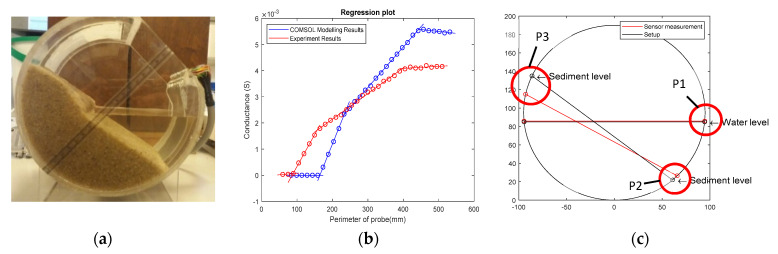
Sensor measured result of partially emergent sediment scenario: (**a**) experiment setup picture; (**b**) plot of sensor’s output conductance vs. probe length; (**c**) plot results of water and sediment surface.

The sensor showed good performance in measuring the depth of two and three phases of media, but limitations must be considered for some specific practical scenarios. Figure 12 shows a scenario where the sediment is only partially immersed in the water; the remainder is above the water surface. In Figure 12c, the red line and black line represent the sensor-measured interface and actual interface, respectively. It was found that the sensor can only detect three interface points: P1, P2, and P3 in Figure 12c. Because the electrical conductivity of non-immersed sediment was non-uniform (being drier further from the water), the interface between immersed sediment and non-immersed sediment was not clear, which caused the left point of the water surface to be difficult to detect. In addition, the demarcation point between sediment and air (P3) has a large error in this scenario, because the dry sediment had a similar conductivity to air. The water surface was assumed to be horizontal in Figure 12c, so that the one interface point for air–water is sufficient to draw the water level. Since the theory of the sensor is based on measuring the different conductivities of media, when two media have very similar conductivity or the media are heterogeneous, the sensor might not detect the interface accurately. Nonetheless, with intelligent interpretation of the data, useful information is still obtained; for example, water level, flow cross sectional area, and that there is emergent sediment are all distinguishable characteristics.

**Figure 13:**
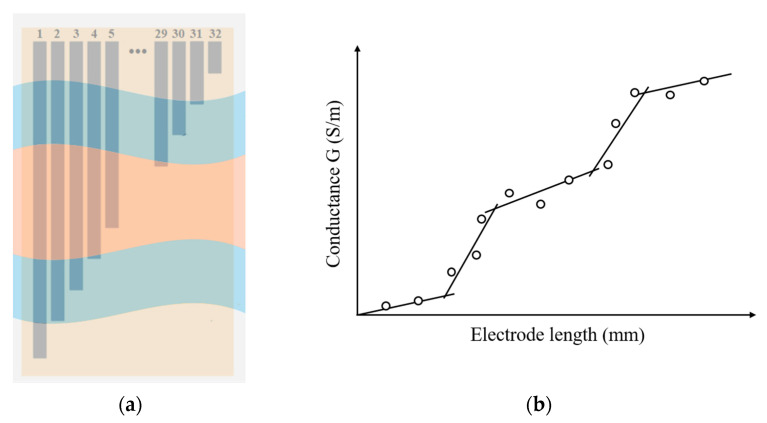
(**a**) Five phase media along the sensor with un-planar interfaces; (**b**) Linear piecewise relationship between conductance and increasing length for un-planar interfaces scenario.

In practical implementation under dynamic conditions, the levels of sediment and water may not be perfectly planar, as shown in Figure 13a. The non-uniform interfaces may lead to different interface levels at different positions across the electrode array; therefore, the measured conductance of each electrode pair could be bigger or smaller than what would be measured with flat interfaces, as shown in Figure 13b. This could influence the detection of interface heights; however, the linear regression process can help to eliminate some of this error. Furthermore, the PCB manufacturing technique can enable electrodes to be very thin and closely spaced (small values of w and d), such that variability across the sensor is minimised. In addition, some interfaces (particularly the interface between air and water) may fluctuate over time due to flow turbulence, which may affect the measurement results. In this study, the data collection from all electrodes can be completed within 0.04 s, meaning an effective sampling frequency of 25 Hz. This frequency could easily be increased for dynamic applications, but nonetheless, turbulent flow surfaces are known to be dominated by frequencies below 10 Hz [31,32,33,34,35,36], so the temporal fluctuation can be accurately captured with the sensor in its current form.

These limitations are relatively minor, especially given the simplicity of the sensor’s structure and application. The sensitivity of the sensor could be improved by optimising the design, for example, decreasing the electrode width, spacing, and length difference. In addition, intelligent approaches such as machine learning could help to interpret ambiguous data objectively and autonomously.

## 5. Conclusions

In this study, a sensor based on conductance has been developed to simultaneously measure the depths of water and sediment in a circular pipe and provide instantaneous monitoring of the interface levels without any calibration process. A theoretical framework was developed and validated. A linear relationship between electrical conductance and electrode length has been observed, and the piecewise linear relationship has been measured and modelled with multiphase media. Most of the results are within the maximum expected error based on the sensor’s resolution. The root mean square error of COMSOL modelling and sensor-measured interface height are within 18% and 33% of the electrode length difference, which is also within 1.4% and 2.6% of sensor’s measurement range, respectively. Furthermore, the experimental and modelled results show that the slope of each sub-line is positively correlated with the electrical conductivity of the measured media and could be used to monitor changes in conductivity. The sensor is best suited to measuring multiphase flows with media that are homogeneous and have appreciably different conductivities. Some limitations have been identified, but these could be overcome by intelligent interpretation of the data, perhaps through a machine learning approach. Ultimately, this sensor provides a new opportunity for low-cost, minimally invasive, and minimally obstructive monitoring of multiphase flows in sewer systems, drainage systems for transport infrastructure, and other similar applications.

## Figures and Tables

**Table 1:** Summary of tests conducted related to the depths of water and sediment, and the rotation angle.

Test Code	Water Depth (mm)	Sediment Depth (mm)	Rotation Angle (Degrees)
W1	134	-	-
W2	95	-	-
W3	47	-	-
S1	133	23	0
S2	135	60	0
S3	133	90	0
S4	94	70	0
S5	94	40	0
S6	95	25	0
S7	48	25	0
S8	47	10	0
S9	48	5	0
SA1	137	56	15
SA2	95	13	28
SA3	47	23	8
SA4	85	75	38

**Table 2:** The electrical properties of materials used in FEA model.

Material	Electrical Conductivity (S/m)	Relative Permittivity
Copper	5.87 × 10^7^	−
Water	0.0284	81
Saturated sand (sediment)	0.0129	15.84
Air	1 × 10^−20^	1

**Table 3:** Depth results summary of water only tests and water and sediment test.

Test Code	Water Depth (mm)					Sediment Depth (mm)				
	Actual	COMSOL Modelling	Model Error	Sensor Measured	Measured Error	Actual	COMSOL Modelling	Model Error	Sensor Measured	Measured Error
W1	134	139.26	5.26	139.35	5.35	−	−		−	
W2	95	92.75	−2.25	93.99	−1.01	−	−		−	
W3	47	44.71	−2.29	46.99	−0.01	−	−		−	
S1	133	132.33	−0.67	138.28	5.28	23	22.34	−0.66	28.72	5.72
S2	135	130.97	−4.03	141.07	6.07	60	59.83	−0.17	55.31	−4.69
S3	133	133.58	0.58	139.28	6.28	90	93.02	3.02	85.89	−4.11
S4	94	92.67	−1.33	99.90	5.9	70	68.73	−1.27	75.92	5.92
S5	94	89.64	−4.36	95.77	1.77	40	36.27	−3.73	35.45	−4.55
S6	95	89.56	−5.44	93.91	−1.09	25	23.05	−1.95	17.37	−7.63
S7	48	47.63	−0.37	48.61	0.61	25	24.82	−0.18	21.74	−3.26
S8	47	45.96	−1.04	47.24	0.24	10	8.72	−1.28	5.99	−4.01
S9	48	46.50	−1.5	46.04	−1.96	5	1.95	−3.05	1.84	−3.16

**Table 4:** Depth results summary of rotated sediment test.

Test Code	Water Depth (mm)				
	Actual	COMSOL Modelling	Model Error	Sensor Measured	Measured Error
SA1	137	136.04	−0.96	138.59	1.59
SA2	95	93.91	−1.09	93.63	−1.37
SA3	47	46.64	−0.36	46.55	−0.45
**Test Code**	**Sediment Depth (Left) (mm)**				
	**Actual**	**COMSOL Modelling**	**Model Error**	**Sensor Measured**	**Measured Error**
SA1	79.00	73.07	−5.93	87.16	8.16
SA2	2.00	3.04	1.04	0.22	−1.78
SA3	33.00	33.74	0.74	36.34	3.34
**Test Code**	**Sediment Depth (Right) (mm)**				
	**Actual**	**COMSOL Modelling**	**Model Error**	**Sensor Measured**	**Measured Error**
SA1	35.33	0.33	27.35	−7.65	35.33
SA2	28.71	−2.29	29.01	−1.99	28.71
SA3	13.71	−1.29	12.43	−2.57	13.71

**Table 5:** Model and sensor measured gradient. The actual conductivity is measured by electrical conductivity meter.

	Actual Conductivity	Model Conductivity	Sensor Measured Conductivity	Actual: Model	Actual: Sensor Measured
Air	0	0.4	0.8	−	−
Water	28	33	23	1:1.18	1:0.82
Sediment	13	15	9.5	1:1.15	1:0.73
